# Seasonal variation in daily activity patterns of snow leopards and their prey

**DOI:** 10.1038/s41598-022-26358-w

**Published:** 2022-12-15

**Authors:** Örjan Johansson, Charudutt Mishra, Guillaume Chapron, Gustaf Samelius, Purevjav Lkhagvajav, Tom McCarthy, Matthew Low

**Affiliations:** 1 Grimsö Wildlife Research Station, Swedish University of Agricultural Sciences, 73993 Riddarhyttan, Sweden; 2 Snow Leopard Trust, 4649 Sunnyside Avenue North, SW, USA; 3 Nature Conservation Foundation, 3076/5, IV Cross Gokulam Park, Mysore, India; 4 Nordens Ark, Åby Säteri, 456 93 Hunnebostrand, Sweden; 5 Snow Leopard Conservation Foundation, 4Th Khoroo, 53-9 Ulan Baatar, Sukhbaatar, Mongolia; 6 Panthera, 8 W 40Th Street, 18Th Floor, NY, USA; 7 Department of Ecology, Swedish University of Agricultural Sciences, 75007 Uppsala, Sweden

**Keywords:** Conservation biology, Behavioural ecology, Animal behaviour

## Abstract

The daily and seasonal activity patterns of snow leopards *(Panthera uncia*) are poorly understood, limiting our ecological understanding and hampering our ability to mitigate threats such as climate change and retaliatory killing in response to livestock predation. We fitted GPS-collars with activity loggers to snow leopards, Siberian ibex (*Capra sibirica:* their main prey), and domestic goats (*Capra hircus:* common livestock prey) in Mongolia between 2009 and 2020. Snow leopards were facultatively nocturnal with season-specific crepuscular activity peaks: seasonal activity shifted towards night-sunrise during summer, and day-sunset in winter. Snow leopard activity was in contrast to their prey, which were consistently diurnal. We interpret these results in relation to: (1) darkness as concealment for snow leopards when stalking in an open landscape (nocturnal activity), (2) low-intermediate light preferred for predatory ambush in steep rocky terrain (dawn and dusk activity), and (3) seasonal activity adjustments to facilitate thermoregulation in an extreme environment. These patterns suggest that to minimise human-wildlife conflict, livestock should be corralled at night and dawn in summer, and dusk in winter. It is likely that climate change will intensify seasonal effects on the snow leopard's daily temporal niche for thermoregulation in the future.

## Introduction

Spatial and temporal niche selection of carnivores and their prey involves trade-offs between the resource gains of food and mates, and the costs of exposure to competitors, predators, hunters or environmental extremes^1–3^. Temporal patterns are most prominent over daily circadian time scales, with species having various anatomical, physiological and behavioural adaptations suited for specific light and temperature conditions (i.e. diurnal vs. nocturnal vs. crepuscular^4,5^). These daily patterns may exhibit seasonality because of seasonal variation in resource distribution or social dynamics, or varying impacts of weather cycles in habitats with summer and winter extremes. Such seasonal changes in daily activity patterns may be reinforced or reduced in response to ongoing climate change^6^.

In predators, particularly large carnivores, activity patterns are commonly expected to follow the movement of their main prey^7–9^, because prey activity is positively correlated to ease of capture^10,11^ or encounter rate^12,13^. However, such correlations are not a necessary condition for predator–prey relationships, since hunting success may be independent of prey activity (with the focus instead being on ‘prey catchability^14,15^). Here, a predator's decision on when to hunt is a trade-off between the encounter rate (related to prey activity) and the probability of successfully catching prey given an encounter. This trade-off is dependent on the predators’ hunting mode, landscape cover and prey behaviour^15–17^. For example, stalk and ambush predators, which largely depend on some form of concealment to approach their prey^14,16^, may use darkness as a substitute for physical cover^18^ and shift from diurnal to nocturnal hunting as habitats vary from closed to open, despite the main activity of their prey being diurnal^19–21^. Under such conditions, the effectiveness of darkness for concealment may decrease in relation to moon luminosity^18,22^, with predator hunting success varying with the moon phase^23^.

While foraging decisions should largely determine the daily activity patterns of large carnivores, one or more of the following factors may force them to adjust when they are active: (1) *Competition—*predators may adjust their temporal activity in the presence of a species with higher competitive ability to avoid kleptoparasitism or intraguild predation^24,25^; (2) *Human activity*—animals may respond to human disturbance and adjust their activity patterns to be less active at times when humans are most active^25,26^; and (3) *Thermoregulation*—animals may shift their activity patterns from nocturnal to diurnal during periods of extreme cold or from diurnal to crepuscular/nocturnal during extreme heat^27,28^. Activity patterns may differ between males and females if factors influence predators in a sex-specific manner, with the most likely differences expected between adult males and females with dependent young.

The snow leopard (*Panthera uncia*) is the apex predator of the mountains of High Asia^29^. An understanding of their daily and seasonal activity patterns, especially how these relate to the activity of their prey and annual temperature cycles, is important for long-term conservation strategies. This is because retaliatory killing by pastoralists in response to livestock predation, emerging infectious diseases, habitat modification, and climate change with increasing temperature extremes during summer are the major conservation challenges they face^29–31^. Despite this, snow leopard activity patterns are poorly understood; current information is limited to VHF-collar tracking^32,33^ and camera trap studies^34–37^. The limitations of VHF-collar technology for monitoring snow leopards is well known^38^ and trap camera studies yield too few encounters to examine daily patterns in detail, especially when considering seasonal variation. This indicates that our knowledge of snow leopard activity patterns and their seasonal variation is not adequate for understanding their ecology, or for effective conservation planning related to predator–prey relationships and disease transmission pathways^29^.

In the mountains of Mongolia, snow leopards primarily prey on Siberian ibex (*Capra sibirica,* 65%) followed by domestic goats (*Capra hircus*) and sheep (*Ovis aries*) (~ 20% combined^39^). However, we currently lack a clear understanding of how snow leopard activity is related to the activity of their main prey, with this limiting our general ecological understanding of the predator–prey relationships and hampering our mitigation efforts targeting livestock-wildlife conflicts^30^. In this study, we use data from GPS-collars with dual-axis accelerometers that provide data on movement in relation to straight-line distance between consecutive GPS locations (displacement movement per unit time) and accelerometer activity (motion sampling at a specific time point). These were fitted to 23 snow leopards (12 males and 11 females), seven Siberian ibex (6 females and 1 male), and 12 domestic goat herds in the Tost Mountains of Mongolia, between 2009 and 2020. From these data, we examine: (1) the general daily activity patterns of snow leopards, and any clear sex-specific differences in their displacement movements and motion sampling, (2) the general daily activity patterns of Siberian ibex and domestic goats, to examine predator–prey activity overlap and whether snow leopard activity can be best explained by prey *activity* or *catchability*, and (3) how seasonality and moon phase influence general daily activity patterns in snow leopards; in particular how daily activity changes between the temperature extremes of high summer and mid-winter.

## Results

### General patterns of movement activity for snow leopards and their prey

**Figure 1:**
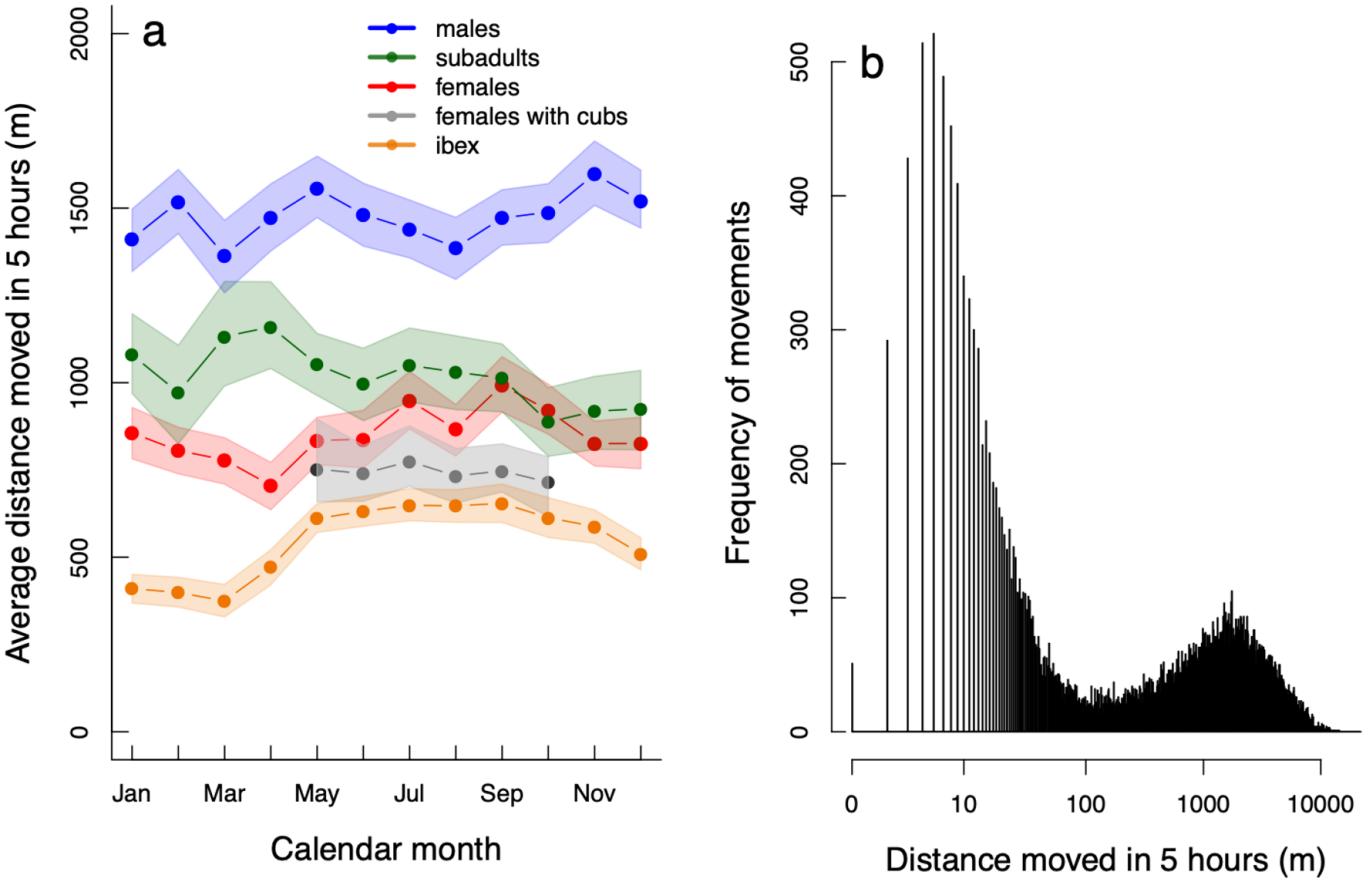
Summary of GPS-movement data: (**a**) mean 5-hourly movement distance (in meters) for each calendar month for snow leopards (adult males = blue, subadults = green, adult females = red, females-with-young-cubs = grey) and for ibex (orange). The monthly estimates were generated from a random-effects GLMM with the joined points representing the mean and the shaded area the 95% CI of the mean. Ibex estimates were from hourly data and were therefore rescaled to be made comparable to the 5-hourly snow leopard estimates. (**b**) Histogram of the log-transformed snow leopard GPS-movement data (male and female adults combined) showing the frequency of observed snow leopard movements related to the distance they moved from one GPS position to the next (5 h later). For separate histograms of males and females see Supplementary Figure S2.

Patterns of average displacement movements for snow leopards were relatively stable across the months of the year (Fig. 1a): with adult males showing the largest average movements estimated over 5-h time periods (mean ± SE of monthly estimates; 1473 ± 92 m), followed by subadults (1017 ± 112 m), adult females (850 ± 95 m) and females with young cubs (750 ± 55 m). Snow leopard females appeared to show longer displacement movements during the warmer months (May–Oct), coinciding with the increased movements of ibex during the same period (Fig. 1a). The frequency of snow leopard movements declined as a function of increasing distance (Supplementary Figure S1). However, by visualising this relationship at the log-scale, it was clear that movements were bimodal for snow leopards; with positional changes made up of short local displacements of up to 150 m, and longer forays of between 100–10,000 m (Fig. 1b; these patterns were similar for males and females: Supplementary Figure S2). For ibex, the log-scale relationship between movement and frequency was unimodal (Supplementary Figure S3), while for domestic goats, the relationship was largely bimodal with a series of shorter displacements (most likely reflecting grazing within pastures) and longer general movements likely related to the herd moving between camps, wells, and pastures (Supplementary Figure S4).

### Daily patterns in movement and motion activity of snow leopards and their prey

**Figure 2:**
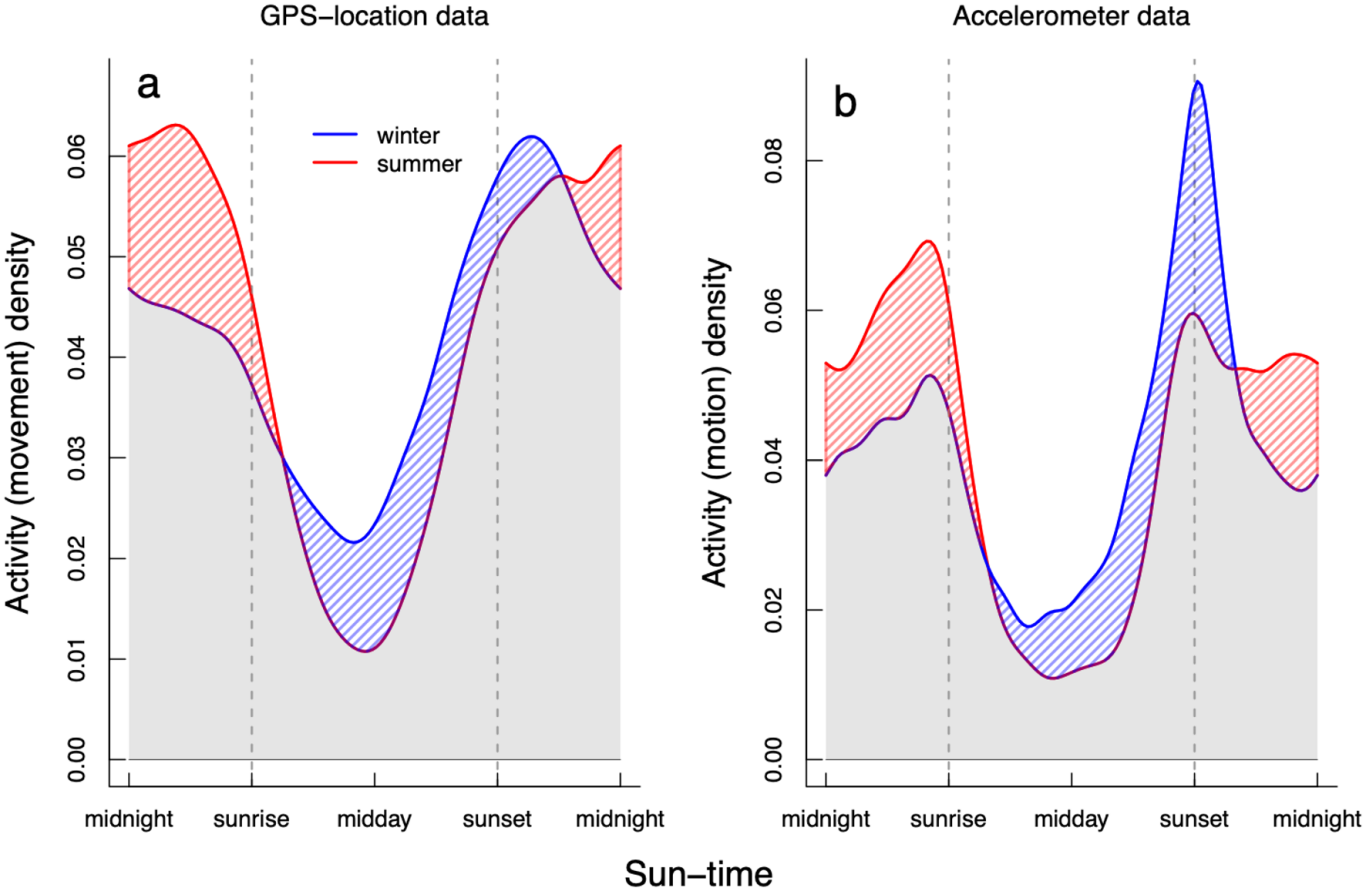
The relative proportion of snow leopard activity across the 24-h cycle for adult males and females during winter (blue) and summer (red) for two types of activity data collected from the GPS-collars (**a** = GPS movement data; **b** = accelerometer activity data). The activity densities on the y-axis have no absolute meaning, but rather are relative measures of probability density calculated from the raw movement data. The time of all observations has been standardised along the x-axis using ‘sun-times’; where the time of observation on each day is calibrated to sunrise, solar-noon (midday) and sunset. These overlap plots show the activity overlap between winter and summer activity (grey) and highlight periods when activity was greater in winter or summer (blue or red shaded polygons, respectively).

Snow leopards showed clear daily activity patterns, with the majority of activity occurring during the night (Fig. 2 & Supplementary Figure S5). From the GPS movement data, there was little evidence of additional crepuscular activity in addition to nocturnal movements; however, the accelerometer motion data indicated that snow leopard activity peaked during sunrise and sunset periods (Fig. 2 & Supplementary Figure S5). There was little difference between the sexes in how they apportioned activity across the day (overlap estimate between males and females = 93%), with the exception of males having a slightly more pronounced difference between night and day activity (proportional movement activity of night [sunset to sunrise] versus day [sunrise to sunset] for males = 66% versus 34%, and for females = 60% versus 40% respectively; Supplementary Figure S5). There was seasonality in these patterns (overlap estimate between summer and winter = 87%), with snow leopards tending to shift their activity towards nocturnal and sunrise periods in summer (i.e. towards the cooler part of the day), and towards daytime and sunset periods in winter (i.e. towards the warmer part of the day; Fig. 2). Thus, snow leopards were more active during the night in summer (proportional movement activity of night versus day = 68% vs. 32%, respectively) compared to winter (58% vs. 42%, respectively). Snow leopard night-time activity was only marginally negatively associated with moon luminosity when examining activity during periods for when the moon was above the horizon (Supplementary Figure S6).

**Figure 3:**
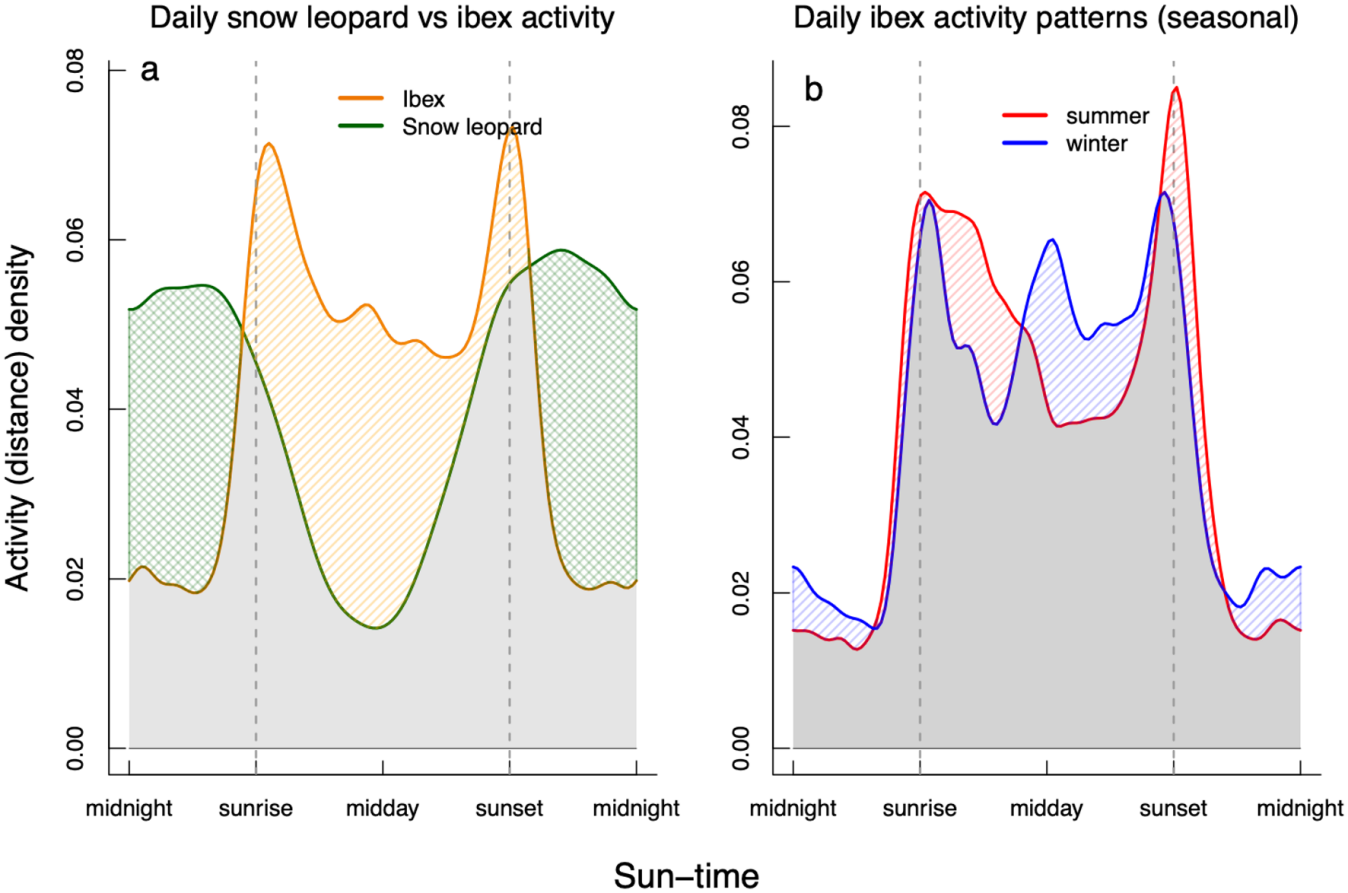
The relative proportion of activity across the 24-h cycle based on GPS movement data for: (**a**) adult snow leopards (green) versus adult ibex (orange), and (**b**) adult ibex during winter (blue) and summer (red). The activity density values have no absolute meaning, but rather are relative measures of probability density calculated from the raw movement data. The time of all observations has been standardised along the x-axis using ‘sun-times’; where the time of observation on each day is calibrated to sunrise, solar-noon (midday) and sunset. These overlap plots show the activity overlap (in grey) between: (**a**) snow leopards and ibex: for periods when snow leopards use more of their activity budget relative to ibex this is shaded (cross-hatched) green, for periods when ibex use more of their activity budget relative to snow leopards is coloured orange. (**b**) Ibex winter and summer activity: for periods when ibex are more active during winter (shaded blue) or during summer (shaded red).

**Figure 4:**
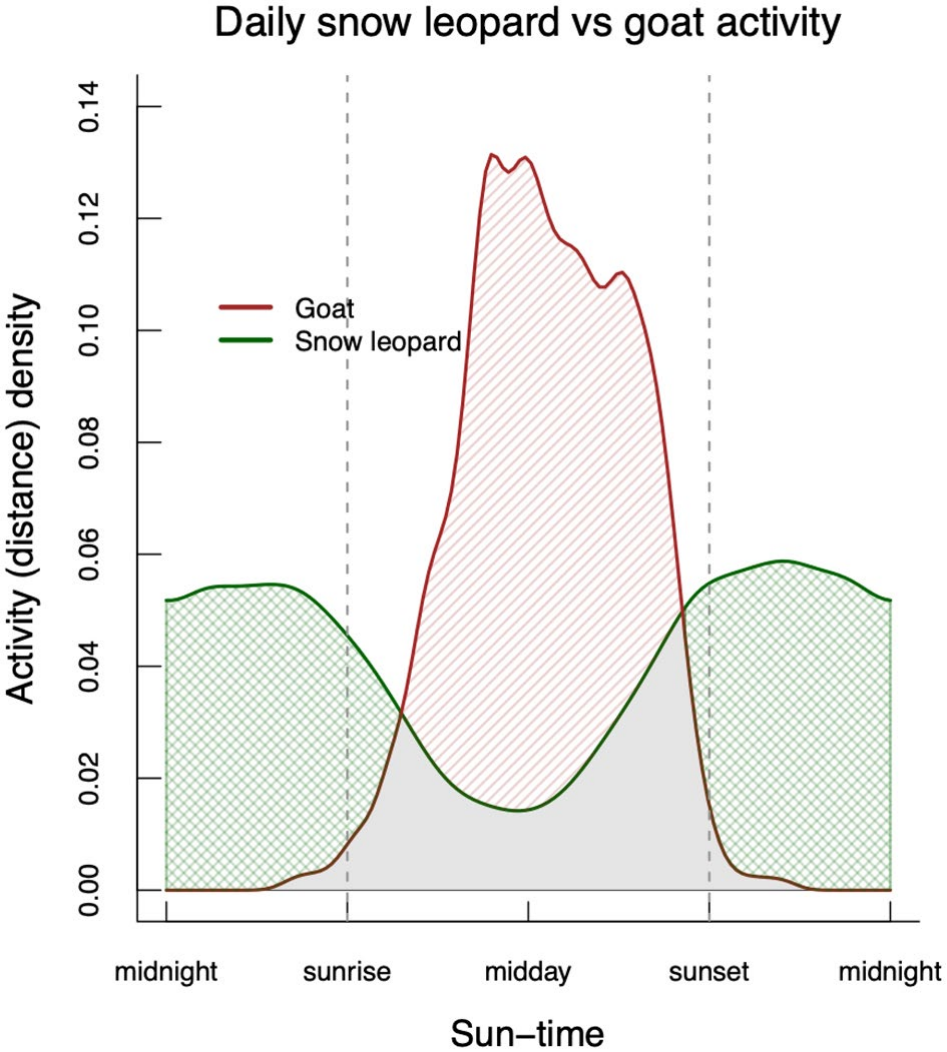
The relative proportion of activity across the 24-h cycle based on GPS movement data for adult snow leopards (green) versus domestic goats (brown). The activity density values have no absolute meaning, but rather are relative measures of probability density calculated from the raw movement data. The time of all observations has been standardised along the x-axis using ‘sun-times’; where the time of observation on each day is calibrated to sunrise, solar-noon (midday) and sunset. These overlap plots show the activity overlap (in grey) between snow leopards and goats: for periods when snow leopards use more of their activity budget relative to ibex this is shaded (cross-hatched) green, for periods when goats use more of their activity budget relative to snow leopards is shaded brown.

Ibex displayed a fundamentally different general pattern in their daily activity compared to snow leopards (overlap estimate between ibex and snow leopards = 66%), with their movements largely occurring during daytime with clear peaks in the morning and evening (Fig. 3a). These opposing patterns of movement activity between ibex and snow leopards could be seen in the proportional activity estimates of night versus day (ibex = 36% vs 64%; snow leopard = 67% vs. 33% respectively). Seasonal change in ibex daily activity was also suggested by the movement data where ibex appeared more active during the mornings and evenings in summer with an additional peak around noon in winter (Fig. 3b). Domestic goats were almost exclusively diurnal in their movement patterns, with little overlap in terms of their general activity patterns compared to snow leopards (Fig. 4).

### Annual cycles of activity of snow leopards during dawn, day, dusk and night

**Figure 5:**
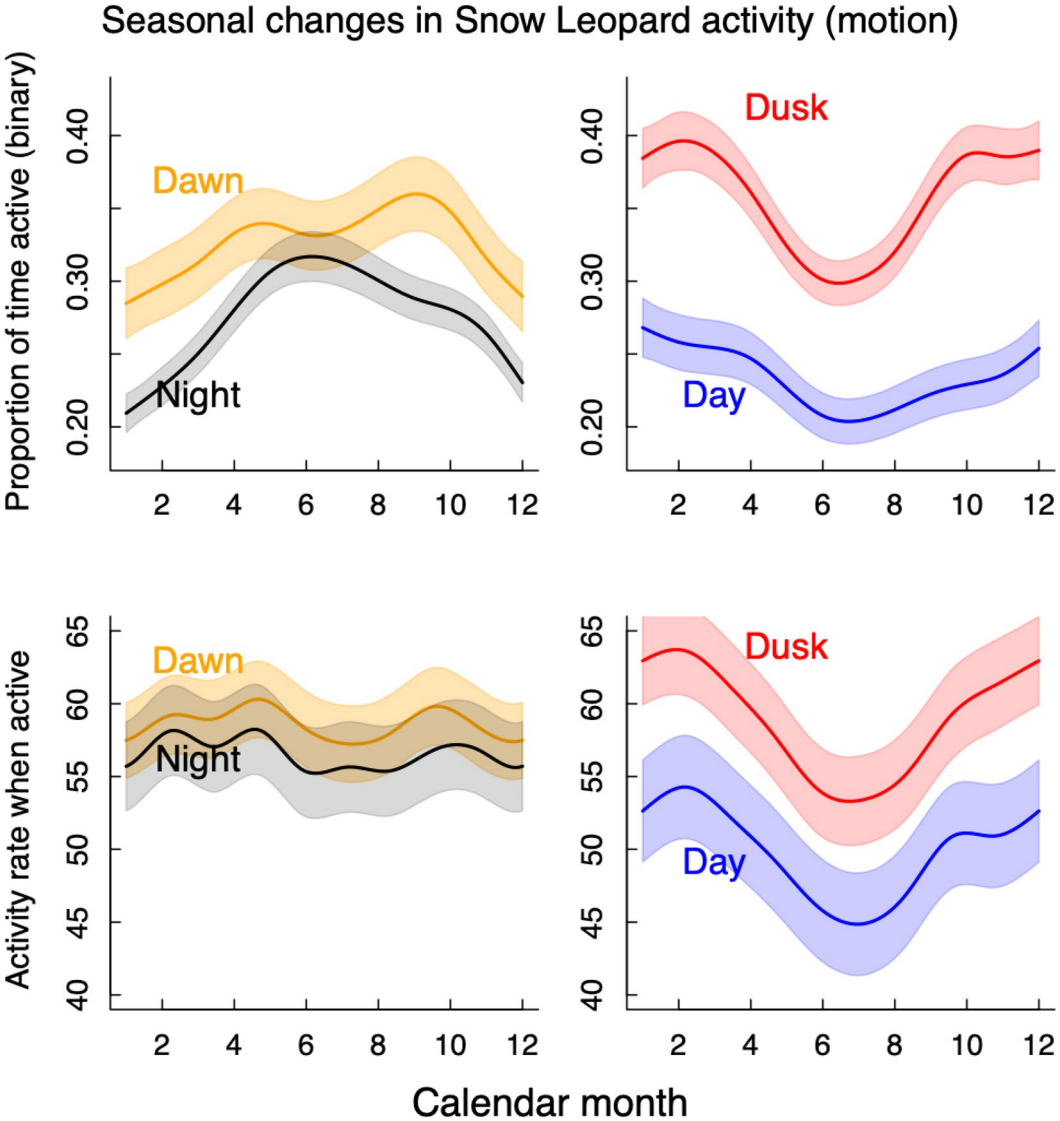
Prediction plots from GAMMs examining variation in snow leopard accelerometer (motion) activity relative to the calendar month for four specific periods during the 24-h cycle: night (black), dawn (yellow), day (blue) and dusk (red). The top two panels show the proportion of total time spent active, and the lower two panels show the activity rate when active. Plots show means (lines) ± SE (shaded areas) generated from the individual models.

The seasonal patterns observed in Fig. 2 were corroborated by the GAMM analyses, which showed clear seasonal changes in snow leopard motion activity during the daily periods of dawn, daytime, dusk and night-time. When categorising these data as a binary indicator of activity (i.e. inactive versus active), night and dawn data showed an increase in the proportion of time spent active during the summer months, while day and dusk showed a decrease in the proportion of time active during the same period (Fig. 5). When considering the activity rate only during times that individuals were active (i.e. excluding the inactive periods), the night and dawn activity patterns did not clearly show the same seasonal trends; however, day and dusk activity rates showed similar seasonal trends of reduced summer activity (Fig. 5).

## Discussion

Snow leopards were mostly active at night and during dawn and dusk, with the degree of crepuscular activity dependent on the method of measurement. Thus, from our results, snow leopards can be generally classified as being facultatively nocturnal with additional crepuscular activity, similar to many other large felids^9,11,40^. However, when examining how the activity patterns changed seasonally, a more complex dynamic emerged (Figs. 2 and 5). We found that snow leopards changed their activity preference between dawn and dusk seasonally. Further, activity patterns at night followed the same seasonal shift as at dawn, whereas daytime activity patterns followed the seasonal shifts of dusk (Fig. 5). Together, these seasonal shifts in activity patterns during different daily time periods suggested that snow leopards adjust their daily activity patterns for thermoregulation by limiting exposure to extreme heat during summer or cold temperatures during winter. The accelerometer motion data and GPS-based movement data showed similar daily patterns (Fig. 2 & Supplementary Figure S5), indicating that accelerometers can be used in place of GPS data for describing general patterns of snow leopard activity.

We found little support for the ‘prey activity’ hypothesis^7,15^ to explain snow leopard activity. Ibex and domestic goats were primarily active between sunrise and sunset, while snow leopards were mostly active between sunset and sunrise (Figs. 3 and 4). Our data suggest that ‘prey catchability’ is a more plausible explanation for higher snow leopard activity during the night in all seasons, as, in an open landscape of high visibility above the tree line, these ambush predators would be better able to approach their prey under the cover of darkness and improve their hunting success^18^. However, this does not explain why peak snow leopard activity often occurred during twilight periods rather than in night-time darkness. We propose two explanations for this. First is that while darkness would increase the likelihood of approaching prey undetected, it may not be ideal for catching the prey during the final ambush if the predator’s vision is compromised by darkness. Thus, higher activity during dawn and dusk might reflect a trade-off in ‘catchability’ between undetected approach, which might be more efficient in darkness, and the final ambush that could be facilitated by more light. This is plausible because the landscape inhabited by snow leopards is typically broken with crevices, rocks and boulders, small bushes and low tufts of grass that provide some cover for a stalking snow leopard, but rarely allow them to approach their prey without crossing open ground. Thus, hunting in these mountains appears similar to the African short-grass savannah, where lions are nocturnal and rely on darkness to successfully attack their primarily diurnal prey^18,22^. The impact of light and vision may be more acute for snow leopards while pursuing prey down steep slopes at high speed. While smaller felid species have slit-like pupils that maximise the amount of light entering the eye during night-time, large felid species have round pupils, which are not as effective at maximising pupillary dilation^41^. Although the limitations of nocturnal vision in large felids have not been quantified, it could explain the peak crepuscular activity patterns found for many of these species^9,40^. Studies examining the prey catchability hypothesis in large felids largely focus on the first part of the hunt, and the need for cover during the approach, but rarely consider the ambush phase^14–16^. If the condition (darkness) that allows an ambush predator like the snow leopard to get close to the prey undetected, subsequently hampers its manoeuvrability or speed during the ambush, then optimal catchability solutions may involve approaching prey under less-than-ideal stalking conditions (e.g. dawn or dusk) as a means to improve hunting success. Thus, the activity patterns we report may reflect poor approach conditions during the day (low activity), excellent approach conditions but only moderate ambush conditions during the night (high activity), good approach conditions and excellent ambush conditions during dawn and dusk (highest activity).

Another plausible explanation for the daily activity patterns of snow leopards could be that they reflect a combination of both the activity and catchability hypotheses. Here, snow leopards would hunt during the night because of the benefits associated with darkness as cover (catchability), and also hunt during the dawn/dusk period because prey is more active than at night and are more likely encountered (activity). Under this scenario there is no need for a trade-off between the success of stalking and the final ambush, as the pattern could be related primarily to the approach phase of the hunt. Both explanations are consistent with our finding that snow leopard night-time activity marginally declined as moon illumination increased. A moonlit night in the vegetation-sparse landscape is relatively bright, meaning that moonlight could be expected to reduce the ability of snow leopards to stalk their prey undetected.

Ambient temperature appeared to have a considerable modifying effect on daily snow leopard activity based on seasonal changes in activity patterns. In summer, when daytime temperatures in the Gobi Desert can reach 40 °C, snow leopards increased their activity during the coolest times of the day (i.e. night and dawn). In winter when night-time and dawn temperatures can reach − 30 °C, snow leopards increased their activity during the warmest parts of the day (i.e. daytime and dusk). Animals are thermoneutral when they do not need to actively regulate their body heat. The thermoneutral range for snow leopards is not known, however, other medium-sized and large felids less adapted to cold climates have a thermoneutral range of 9 to 37 °C ^28^. The Arctic fox (*Vulpes lagopus*)*,* a species well-adapted to polar conditions, are thermoneutral down to − 7 °C with their winter coat and 5 °C in summer^42^. We expect that winter temperatures in the Gobi Desert commonly fall below the lower thermoneutral temperature for snow leopards, and climb above this range in summer. Seasonal changes in snow leopard activity seemed to be in response to their need for regulating their body temperature. Wind has a dramatic effect on body temperature maintenance in snow leopards (Ö Johansson, personal observation), and thus by being more active during the warmer daytime periods in winter and increasing their sheltering during the coldest times, it is likely snow leopards were conserving energy. In summer, the daytime temperatures in the high-altitude and vegetation-sparse mountainous habitat are exacerbated by the intense solar radiation. With limited water sources in the Gobi, which possibly limits their ability to pant as the principal method used by felids to cool down^43^, adjusting activity to cooler time periods in summer presumably allows snow leopards to conserve energy and water, and avoid hyperthermia. Similarly, the ibex in our study area also appeared to adjust activity patterns for thermoregulation during daylight hours; in summer, activity increased in the morning compared to an increase in activity in the afternoon and evening in winter. We expect that with temperatures in High Asia increasing at a higher rate than the global average in recent years^31^, snow leopards will likely respond to future changes by further restricting their daytime and early evening activity during summer months; such information will be important for modelling how climate change may impact snow leopard and prey distributions^44^.

We found no evidence of snow leopard activity being impacted by human activity in our study area. If snow leopards were primarily avoiding humans, we would expect daytime activity to be lower in winter when the human and livestock density in our study area is higher in this area: but we observed the opposite pattern. The mountainous habitats and low human density (typically < 1 person per sq. km) likely allow snow leopards to effectively avoid humans spatially regardless of human activity patterns, because people rarely frequent the very steep and rugged parts of this environment that constitute snow leopard habitat. This is supported by Sharma et al.^45^ who found no influence of human population density on activity patterns of snow leopards as indicated in camera trap data. Despite this, retaliatory killing of snow leopards by local people in response to livestock predation (particularly goats) remains an important threat across the snow leopard's range^30^. In order to mitigate such conflict, a better understanding of spatial and temporal patterns of livestock predation by snow leopards is needed to supplement the activity data collected in this study. Based on these activity patterns, we predict that livestock are most vulnerable to snow leopard predation during the last hours of the night or dawn in summer, and the dusk and early hours of the night in winter. This suggests that herders in snow leopard habitat be advised to ensure their livestock are corralled in predator-proof enclosures^46^ or carefully supervised during these periods.

## Methods

### Study area

Our study was conducted in Tost Mountains (43°N, 100°E), of the Gobi Desert in the southernmost part of Mongolia between 2009 and 2020. The mountains consist of several rugged massifs (altitude 1600–2500 m above sea level), crossed by steep ravines and separated by wider valleys. As is typical of snow leopard habitats, the landscape is very open with sparse vegetation, consisting mainly of short grasses, dwarf shrubs and patches of shrubs. Common plants include *Amygdalus mongolica, Stipa* spp., *Caragana leucophlaea* and *Eurotia ceratoides*. Overcast weather is rare (< 55 days per year, www.worldweatheronline.com), implying a large difference in illumination between moonless nights and nights with full moon. The low heterogeneity in vegetation potentially increases the importance of darkness as a reliable source of concealment and hunting cover. The climate is windy and dry with < 130 mm precipitation annually, of which approximately 70% falls as rain from June–August. Seasonal variation in temperature in our study area was extreme, with mean daily minimum and maximum temperatures at the eastern end of the study area (1650 m.a.s.l.) being − 27 °C and 1 °C in January, and 11 °C and 33 °C in July, respectively. This allows us to assess the influence of thermoregulation on daily activity by examining seasonal changes in these daily patterns. We expected low intra-guild influences on activity patterns of snow leopards because the only other large carnivore in the study area, the wolf (*Canis lupus*), use the steppes and relatively less rugged terrain compared to those preferred by snow leopards and ibex^37,47^. Livestock herding was the only livelihood within the study area. Livestock and herder density in the mountains varied from ~ 10 herder families in summer to ~ 80 herder families in winter. Herded livestock, goats and sheep, are accompanied by a herder while grazing and penned at camp sites at night^46^.

### Data collection: Snow leopards

Twenty-three snow leopards (11 female, 12 male) were captured between 2009 and 2019 with modified Aldrich style snares set at their marking sites and immobilised with a combination of tiletamine-zolazepam and medetomidine^48,49^. Captured animals were fitted with GPS-collars programmed to record the animal’s location every fifth hour (GPS Plus and Vertex Lite, Vectronic Aerospace, Gmbh, Berlin, Germany), and also contained two single-axis accelerometers, acquiring instantaneous activity data on lateral (y-axis) and posterior-anterior (x-axis) movements. The accelerometers measured movements four times per second and logged mean values ranging from 0 (immobile) to 255 (very active) every five minutes. Location data were retrieved daily via satellite uplink, while accelerometer activity data were accessed once the collar was retrieved, either when the set-time drop-off released the collar (after 12–22 months), during a re-capture event, or in case a snow leopard died. The GPS data were subsequently standardised by removing erroneous locations and outliers, following the methodology in Bjørneraas et al.^50^ with metrics adjusted for our data (*D* = 100 000 m; *m* = 25 000 m; *a* = 5000 m/h; *q* =  − 0.97), and by removing locations falling outside the 5-h interval ± 15 min. Because female movements are restricted in the first months after parturition^51^, we classified females as being with small cubs from time of birth until cubs were five months old and removed data from these females from the general activity data analyses. In the distance analyses, these data were treated as a separate group.

We collected two types of activity data from the snow leopard collars: (1) accelerometer data that provided finer-detailed ‘motion’ activity information; these data consisted of 2,329,450 measurements collected from 17 individuals (ten males and seven females; because six collars have not been recovered yet) over a total of 8927 collar-days from April 2010 to Jan 2020 (mean ± SD = 470 ± 187 days of 5-min data/individual), and (2) GPS-location data from which we calculated straight-line distance between consecutive locations (displacement) as a measure of ‘movement’ activity; these data consisted of 34 959 GPS locations, collected over 12,315 collar-days from June 2009 to February 2020 (*n* = 23 individuals; mean ± SD = 1524 ± 906 locations/individual). We used both datasets to examine daily activity patterns; the accelerometer data representing fine-scaled motion to better capture daily variation, and the GPS-data allowing comparison with similar datasets on the ibex and domestic goat (see below). Also, by comparing the two types of data we could better interpret how the motion activity data reflected actual displacement movements of the animals across the landscape.

### Data collection: Ibex

Seven ibex (6 females, 1 male) were free-darted at a water-hole, using a combination of ketamine (2.8 mg/kg) and medetomidine (0.20 mg/kg) administered with a CO_2_-powered dart rifle (Daninject J.M. Special, Daninject, Borkøp, Denmark). The capture crew consisted of a darter in a blind approximately 20 m from the water-hole and a spotter on a mountainside 300 m away. The spotter provided information to the darter and monitored the darted ibex during induction. Captured animals were fitted with GPS-collars (GPS Vertex Lite, Vectronic Aerospace, Gmbh, Berlin, Germany) programmed to take a GPS location every hour and accessed via daily satellite uplink. These location data allowed calculation of straight-line movements between consecutive locations as a measure of displacement movement activity; these consisted of 42 832 GPS locations, collected over 1727 collar-days from April 2018 to May 2020 (*n* = 7 individuals; mean = 5904 locations/individual; range 1067–13 326).

### Data collection: Domestic goats

One goat from each of 12 herds in the central part of Tost, the area with the highest snow leopard density, was fitted with a GPS-collar (GPS Vertex Lite, Vectronic Aerospace, Gmbh, Berlin, Germany) from November 2019 to November 2020. Collars were programmed to take a GPS location every hour from 07:00 to 22:00, to capture the times when the animals were outside their nighttime corrals and moving^46^. An additional location was collected at 03:00 to confirm that the animals were confined during the nighttime. These data generated 68 875 GPS locations collected over 4054 collar-days (mean 5740 locations / individual, range 3 957–6 177).

### Ethics

We obtained all relevant permits from Mongolia’s Ministry for Environment and Green Development to allow us to conduct research, capture and collar snow leopards, ibex and goats in our study area. All animal handling followed the appropriate ethical standards and were carried out in accordance with relevant guidelines and regulations. These methods were approved by the Board for Animals in Research and Teaching at the Swedish University of Agricultural Sciences (permits SLU-ua-2020.4.1–2294 and SLU-ua-2020.4.1–2295). The capture teams consisted of highly experienced staff with appropriate training and equipment. We employ a custom-made surveillance system that monitors our traps, ensuring that animals spend as little time as possible in the traps^49^. All capture, handling and monitoring of animals in this study follow rigorous ethical guidelines as detailed in^49^. Methods in this paper are reported in accordance with ARRIVE guidelines^52^.

### Analyses

For all analyses, we removed data collected during the first 24 h to avoid any influence of the capture. We also excluded accelerometer (motion) activity data for periods when snow leopards were presumed to be on kill sites: i.e. when four or more consecutive locations were within 200 m, because snow leopards commonly remain inactive, usually within 20 m of the kill, for several days; or when mothers had young cubs (up to the age of 5 months). Motion activity analyses used here are from the x-axis data only, since activity values on x- and y-axes were highly correlated (Pearson’s correlation, *r* = 0.96) and y-axis values produced virtually identical results. Analysis using accelerometer data when we categorised motion activity as either ‘resting’ or ‘active’ are based on behavioural observations on a captive snow leopard^53^, and a GPS-collar study on the Eurasian lynx (*Lynx lynx*^9^;), activity values < 28 were classified as resting and values ≥ 28 were considered active. Snow leopards were classified as adults or subadults following Johansson et al.^54^, based on separation from their mother at 20–22 months^55^. During the study, two males and three females transitioned from subadult to adult and were reclassified accordingly.

We first characterised broad annual patterns of activity by comparing monthly variation in the 5-hourly GPS-location movement data between adult males, adult females, females-with- young cubs and subadult snow leopards. This was based on visually describing the raw movement data for each group, and estimating the between-group differences using Bayesian Generalised Linear Mixed Models (GLMMs) where both month and individual identity were included as random effects (for the formal model description and analytical details see Supplementary Methods).

We then examined daily variation in motion (accelerometer) and movement (GPS) activity patterns of snow leopards. Because the snow leopard GPS-movement data were recorded 5-hourly, we used these data to create an average movement observation for each of the 5 h that the period encompassed (i.e. 1/5 of the total movement was assigned to each period hour). To check that these data would capture the general variation in daily activity patterns we were interested in, we simulated similar data and then ‘sampled’ this movement every 5 h and back transformed it into hourly observations. This demonstrated that if many samples are taken, this method of estimation largely preserves the original pattern of daily variation (with minor ‘blunting’ of peaks and troughs; see Supplementary Methods). For the activity patterns, we were particularly interested in how activity varied in relation to light intensity of the sun: so-called ‘sun time’ rather than clock time. It has been clearly shown that for animals with diurnal variation in activity patterns in seasonal environments, times of observation should be converted to ‘sun times’ so that all observations across the year are calibrated to sunrise, sunset and the sun’s zenith^56^. To do this we used the sunTime() function from the ‘overlap’ package in R^57^. This uses GPS positional information and the date of observation to adjust clock time to sun times for all observations. Because we were primarily interested in describing these activity patterns relative to daylight intensity, seasonal variation, and making comparisons between snow leopard, ibex, and goat, we used a method to create daily activity density curves: i.e. rescaling the y-axis so that it represented a relative measure of activity where the area under the daily activity curve = 1. The advantage of this approach is that it allowed us to directly compare the proportion of activity occurring during specific periods and overlap between different seasons, sexes or species. To do this we wrote a simple Metropolis–Hastings Markov chain Monte Carlo algorithm in R^58^ that used mean estimates from the raw data to probabilistically sample from the shape of the distribution that best approximated the daily activity patterns for the group we were interested in (Supplementary Methods). These 10,000 samples (separately generated for each group) were then used to generate activity density plots and between-group overlap calculations and plots using the ‘overlap’ package; Supplementary Methods). The between-group comparisons focused on comparing daily activity patterns (both motion and movement) of snow leopard females versus males and snow leopard activity in summer (May-Aug) versus winter (Dec-Feb). We also described the daily activity patterns of ibex and domestic goats, and compared these patterns based on GPS-movement data to snow leopards. The ibex and goat displacement movement data were converted into activity density data in the same way the snow leopard data were treated. For determining proportional comparisons between day and night, we counted the number of samples that occurred between sunrise and sunset (π/2 and 3*π/2 radians of ‘suntime’) and divided this number by the total number of samples taken (i.e. 10,000).

Finally, we examined how snow leopard activity during different key periods of light intensity during the day (i.e. dawn, day, dusk and night) changed across the annual cycle, as a further means of examining variation in daily activity associated with seasonality. All activity data were classified as dawn, day, dusk or night, where we defined time periods based on astronomical twilight, which is when the centre of the sun is between 0 and 18 degrees below the horizon. Dawn was defined as beginning of astronomical twilight to sunrise, day as sunrise to sunset, dusk as sunset to the end of astronomical twilight and night as the period between twilights. Data on sunrise, sunset, moonrise and phase were obtained from the United States Naval Observatory (http://aa.usno.navy.mil.data/). Seasonal changes in the activity patterns during these periods were analysed and visualised using generalised additive mixed models (GAMMs) in the R package ‘mgcv’^59^. We included month as a smoothed fixed effect to examine how snow leopard motion activity changed in each of these periods based on two different activity components: (1) the proportion of time active (i.e. proportion of accelerometer measurements ≥ 28), and (2) the measure of accelerometer activity when active (i.e. accelerometer values between 28 and 255). We also included individual snow leopard ID as a random effect in these models to control for uneven sampling between individuals. For night activity data, we estimated lunar illumination based on moonrise and phase, and used similar GAMMs to examine whether motion activity in snow leopards was related to the extent of lunar illumination (as a smoothed fixed effect; see Supplementary Methods for GAMM model formulations).

## Supplementary Information

Supplementary Information 1.

Supplementary Information 2.

Supplementary Information 3.

Supplementary Information 4.

Supplementary Information 5.

## Data Availability

The GPS location data for collared snow leopards are judged to be highly sensitive data in the context of their conservation and are not available for general distribution. However, the displacement movement data derived from these raw data that were used to calculate the daily activity patterns in this paper, which includes an individual animal identifier, its basic demographic data, the local time of sampling its position, and displacement movement since the previous measure are available in the supplementary material (Supplementary Information 1-4) included with this paper.
