# Supplementary material for: Seasonal variation in daily activity patterns of snow leopards and their prey
Source: Sci Rep. 2022 Dec 15;12:21681. doi: 10.1038/s41598-022-26358-w (PMC9755138; doi:10.1038/s41598-022-26358-w)
Supplement: Supplementary file 4 — Supplementary Information 4. [file 41598_2022_26358_MOESM4_ESM.pdf]

Displacement Movement Data used in the paper:

Seasonal variation in daily activity patterns of snow leopards and their prey

Included are three data files (csv format) containing the following information:

File 1: Displacement activity data Snow Leopards has 6 columns with the individual ID, the sex of the individual animal, age (adult vs sub-adult), if it is a female with young cubs >5 months old (1 = yes, 0 = no), the displacement movement from the previous GPS location taken 5 hours ago, and the local date and time at the time of the current GPS location.

File 2: Displacement activity data Ibex has 3 columns with the individual ID, the local date and time at the time of the current GPS location, and the displacement movement from the previous GPS location taken 1 hour ago.

File 3: Displacement activity data Domestic Goat has 3 columns with the individual ID, the local date and time at the time of the current GPS location, and the displacement movement from the previous GPS location taken 1 hour ago.
