# Supplementary material for: Seasonal variation in daily activity patterns of snow leopards and their prey
Source: Sci Rep. 2022 Dec 15;12:21681. doi: 10.1038/s41598-022-26358-w (PMC9755138; doi:10.1038/s41598-022-26358-w)
Supplement: Supplementary file 5 — Supplementary Information 5. [file 41598_2022_26358_MOESM5_ESM.pdf]

Supplementary Data for the paper:

Seasonal variation in daily activity patterns of snow leopards and their prey

Örjan Johansson, Charudutt Mishra, Guillaume Chapron, Gustaf Samelius, Purevjav Lkhagvajav, Tom McCarthy & Matthew Low

Included in this material:

Supplementary Methods:

A. Model formulation for the GLMMs and GAMMs used in the analyses

B. Converting the 5-hour GPS movement data to hourly movement data

C. Metropolis-Hastings Markov chain Monte Carlo algorithm in R

Supplementary Figures:

S1: Histograms of the snow leopard data (raw)

S2: Histograms of the log-transformed data snow leopard data (females and males)

S3: Histogram of the log transformed ibex displacement movement data

S4: Histogram of the log transformed domestic goat displacement movement data

S5: Sex disaggregated snow leopard activity data (movement versus motion)

S6: Nocturnal motion activity relative to the moon illumination fraction

Supplementary Methods A: Model formulation for the GLMMs and GAMMs used in the analyses

GLMM LogNormal model used to estimate the means and 95% CIs of the movement estimates for each of the groups displayed in Figure 1 (snow leopards: adult male, adult female, subadult, females-with-young-cubs and ibex). Here 'i' refers to each observation, 'j' is the grouping variable for the number of individuals (i.e. 23 for adult snow leopards and 7 for ibex), and 'k' the grouping variables for month (i.e. n = 12). All monthly outputs from this model (shown in Fig. 1) were derived by adding the global intercept to the estimated monthly 'error' to generate monthly means and 95% CIs from the derived posterior distribution.

#GLMM general model formulation

$\log(\text{Movement}_i) \sim \text{Normal}(\mu_i, \sigma)$

$\mu_i = \alpha_{global} + \varepsilon_{individual\ j} + \varepsilon_{month\ k}$

$\varepsilon_{individual\ j} \sim \text{Normal}(\mu_{individual}, \sigma_{individual})$

$\varepsilon_{month\ k} \sim \text{Normal}(\mu_{month}, \sigma_{month})$

#priors

$\sigma \sim \text{Gamma}(0.001, 0.001)$

$\alpha_{global} \sim \text{Normal}(5, 100)$

$\sigma_{individual} \sim \text{Uniform}(0, 5)$

$\sigma_{month} \sim \text{Uniform}(0, 5)$

$\mu_{individual} \sim \text{Normal}(0,100)$

$\mu_{month} \sim \text{Normal}(0,100)$

Because these GLMMs were formulated in a Bayesian framework, we performed model checks for convergence of the chains (by visualizing the stability of individual chains and the mixing of multiple chains for the final estimation), and the appropriateness of the likelihood distribution and model formulation by undertaking poster predictive checks where we simulated data from the model and compared the means and variances to the raw data. In all cases the Bayes P values were between 0.4 & 0.6 (ideal range is between 0.1 & 0.9). These were implemented in JAGS (Plummer 2007) run from R (R Core Team 2019).

GAMM models for snow leopard activity during different time periods

We ran two types of GAMMs on the accelerometer activity data from the snow leopards to examine seasonal changes in general activity during four periods during the 24-hour cycle: dawn, day, dusk and night. For all GAMMs we used month as a fixed smoothing term to explain variation in the activity rate and included the individual ID as a random effect in the gamm() function from the 'mgcv' package in R.

The general model structure for the activity data structured as a binary variable to look at patterns of activity versus inactivity (i.e. the proportion of time active as defined as accelerometer values of <28 versus >28) was:

$\text{Activity}_i \sim \text{Binomial}(p_i)$

$\text{logit}(p_i) = \alpha_j + s(\text{month}_i)$

$\alpha_j \sim \text{Normal}(0, \sigma)$

The general model structure for the activity data when only looking at activity when the animal was not resting (i.e. accelerometer values of >28; see *Nygren, E. 2015. Activity patterns of snow leopards (Panthera uncia) at their kill sites. Master's thesis, Swedish University of Agricultural Sciences, Uppsala, Sweden*) was:

$\text{Activity}_i \sim \text{Normal}(\mu_i, \sigma)$

$\mu_i = \alpha_j + s(\text{month}_i)$

$\alpha_j \sim \text{Normal}(0, \sigma_{\text{individual}})$

For activity data collected at night we also estimated lunar illumination based on moonrise and phase, and used GAMs to examine whether motion activity in snow leopards was related to lunar illumination during the night periods when the moon was above the horizon (as a smoothed fixed effect).

$\text{Activity}_i \sim \text{Normal}(\mu_i, \sigma)$

$\mu_i = \alpha_j + s(\text{month}_i)$

$\alpha_j \sim \text{Normal}(0, \sigma_{\text{individual}})$

Supplementary Methods B: Converting the 5-hour GPS movement data to hourly movement data

Because the snow leopard GPS-movement data was taken 5-hourly, we used these data to create an average movement observation for each of the 5 hours that the period encompassed (i.e. 1/5 of the total movement was assigned to each period hour). To check that these data would capture the general variation in daily activity patterns we were interested in, we simulated similar data and then ‘sampled’ this total movement every 5 hours and back transformed it into hourly observations. This demonstrated that if many samples are taken, this method of estimation largely preserves the original pattern of daily variation, especially when patterns shift between periods of stable activity (Fig. A). However, when there are sudden changes of large magnitude or short-term peaks and troughs in the activity patterns, these effects may be ‘smoothed’ in appearance (Fig. B) or ‘blunted’ in their magnitude (Fig. C).

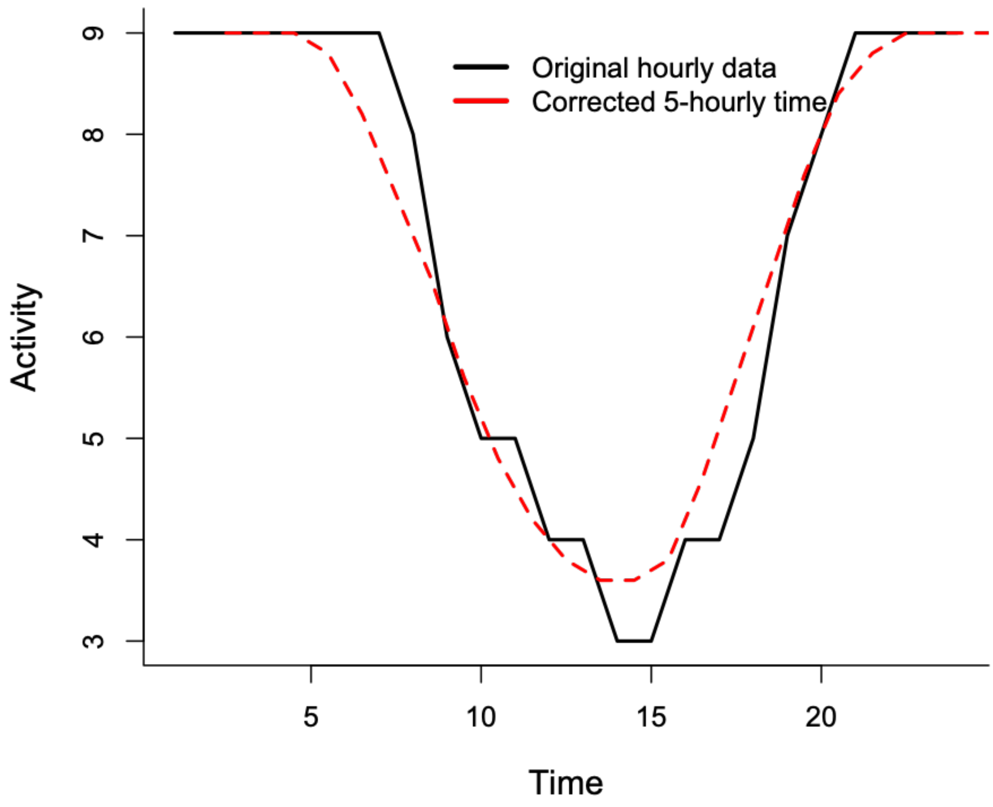

Fig. A: Comparing the temporal patterns of simulated activity data collected hourly (in black) and the same data if collected 5-hourly and then ‘back transformed’ into hourly data by assigning 1/5 of the 5-hourly measurements to the 5 hourly periods that comprised that 5-hourly measurement (see methods)

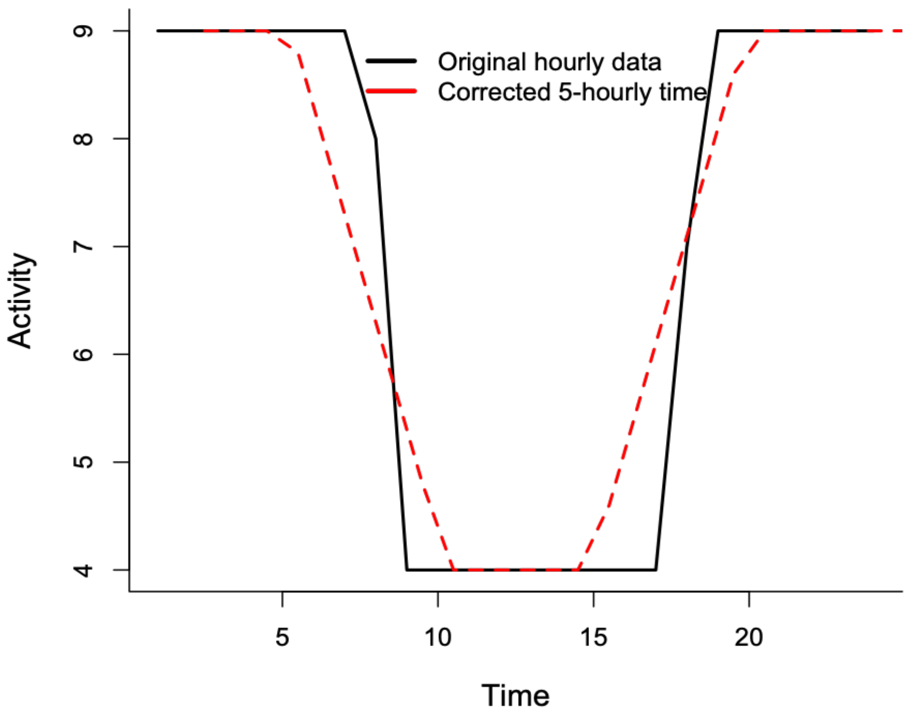

Fig. B (above) and Fig. C (below). Showing the same relationships as in Fig. A, but with different patterns in the original activity data.

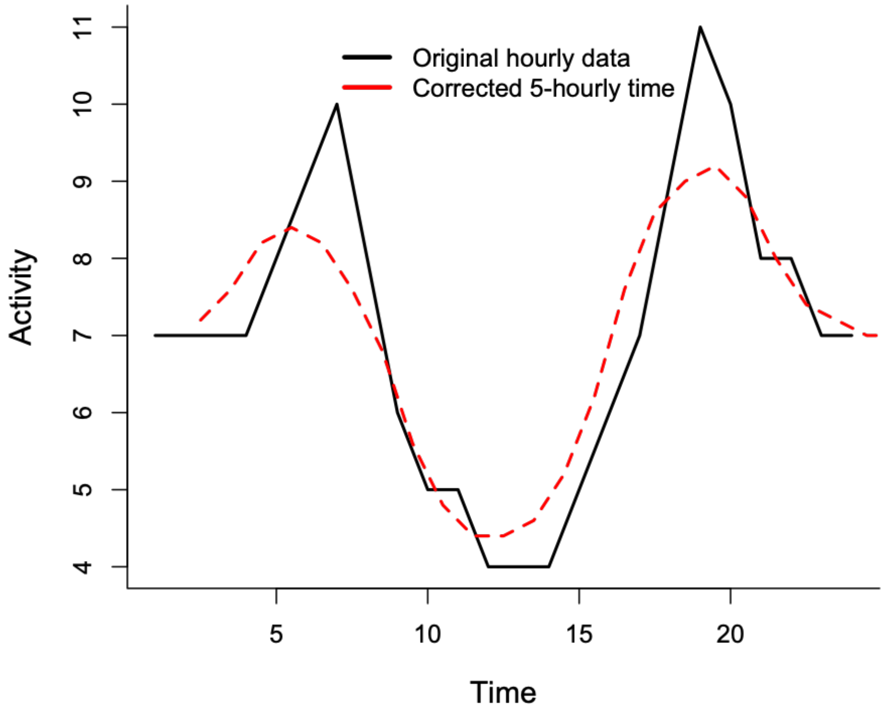

Supplementary Methods C. Metropolis-Hastings Markov Chain Monte Carlo algorithm in R

This code was used to probabilistically sample from the shape of the distribution that best approximated the daily activity patterns for the group we were interested in (i.e. mean estimates of activity in relation to ‘sun times’ from the raw data. The result of the 10,000 MCMC samples generated from this algorithm is that they could then be used to generate activity density plots and between-group overlap calculations and plots using the ‘overlap’ package in R (overlapPlot and overlapEst functions; Ridout & Linkie 2009). This sampling method also allows proportional estimates of total activity during different time periods, in the same way that probabilities can be calculated from Bayesian posterior distributions.

```
#read in the data
data<-read.csv("suntime_activity_data.csv")

#here there are two columns
data$sun.times #where the observation time of the acvtivity is recorded in 'sun.time' (see Nouvellet
 et al 2011)
data$activity #the activity level (either movement or motion) as recorded by the GPS collar for that
 corresponding 'sun.time' period

#create a series of 60 activity means spread across the range of suntimes spanning the day
#can choose as many as you want, but 60 seems to be a good compromise between daily pattern
 detail and data availability at each break point

breaks=60
range<-seq(min(data$sun.times), max(data$sun.times), length.out=breaks+1)

mean.out<-c()
for(i in 1:breaks){
 where<-data$sun.time>=range[i] & data$sun.time<range[i+1]
 mean.out[i]<-mean(data$activity[where], na.rm=T)
}

#now take these measures of daily activity and convert this into a probability density using a simple
 Metropolis-Hastings type sampler
#these data can then be used for density plotting and overlap plotting using the overlap package

sun.time=range[-(breaks+1)] #values of x (sun times)
y=mean.out #values of y (activity)

samples <- 10000 #number of MCMC samples
chain.values <- numeric(samples) #where to store the chain
x.position<-1:length(sun.time)
activity<-y
```

```
current.chain.position <- sample(sun.time,1) #random starting location


for(i in 1:samples){

#record current x (sun.time) value
chain.values[i] <- sun.time[current.chain.position]

#randomly decide another sun.time value to visit (excluding the current value)
proposal <- sample(x.position[-current.chain.position], 1)

#decide if move or stay
prob_move <- activity[proposal]/activity[current.chain.position]
current.chain.position <- ifelse(runif(1) < prob_move, proposal, current.chain.position)

}

#now the vector 'chain.values' has 10,000 sun.time values in it, with the number of specific sun.time
 values representing the proportional value of activity in the same shape as the original data. This
 can be used for density plots and for calculating the proportional  activity within different time
 periods
```

Supplementary Figure S1. Histograms of the snow leopard data (raw)

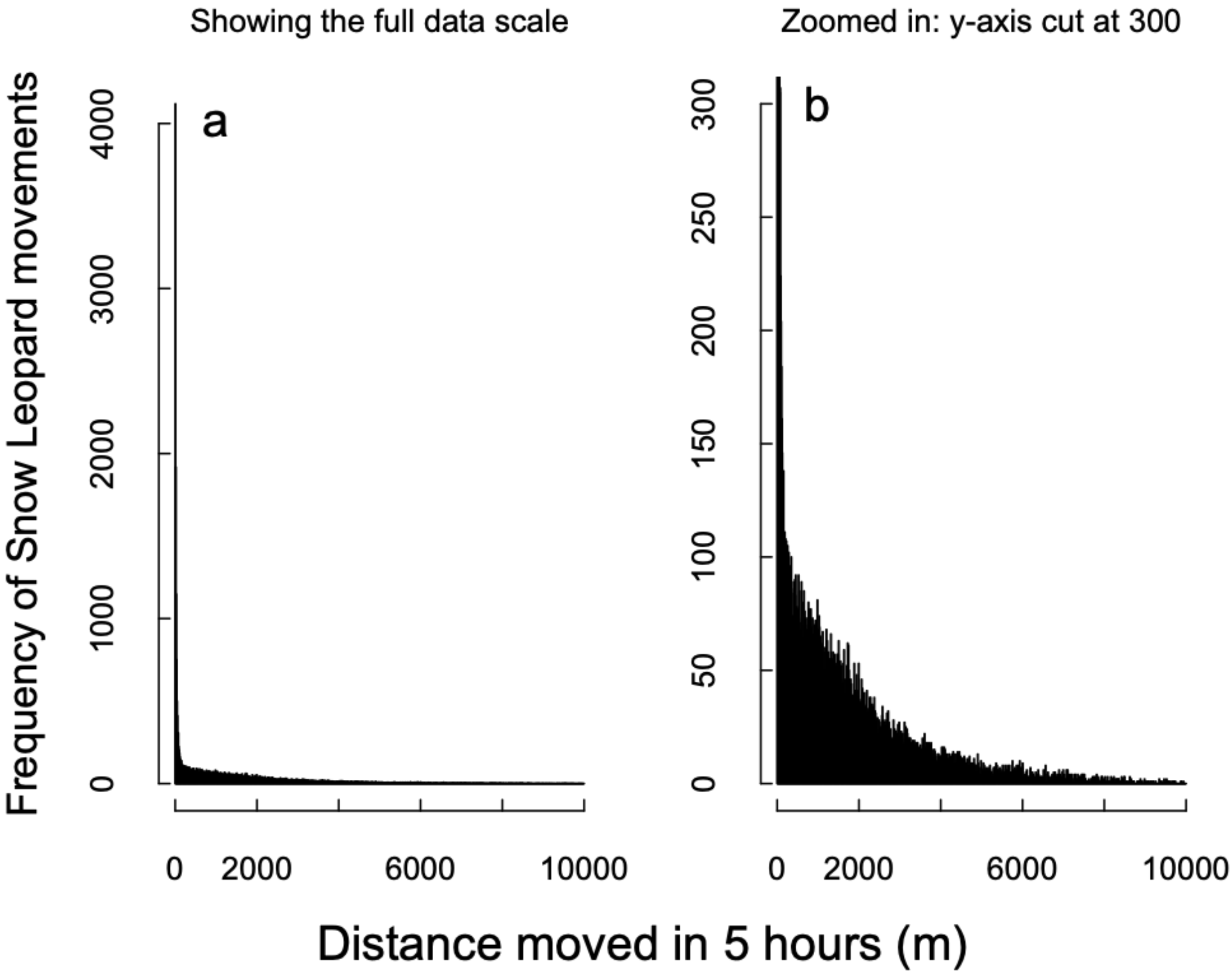

Fig. S1. Histogram of the raw snow leopard GPS-movement data showing the frequency of observed snow leopard movements related to the straight-line distance (displacement) they moved from one GPS position to the next (5 hours later). The left panel (a) shows all the data, but because of the extreme number of points close to zero, the rest of the data are difficult to see at that scale. The right panel (b) is the same figure, but the y-axis has been truncated at 300 to better show the patterns of the data distribution for distances greater than 50m.

Supplementary Figure S2. Histograms of the log-transformed data snow leopard data

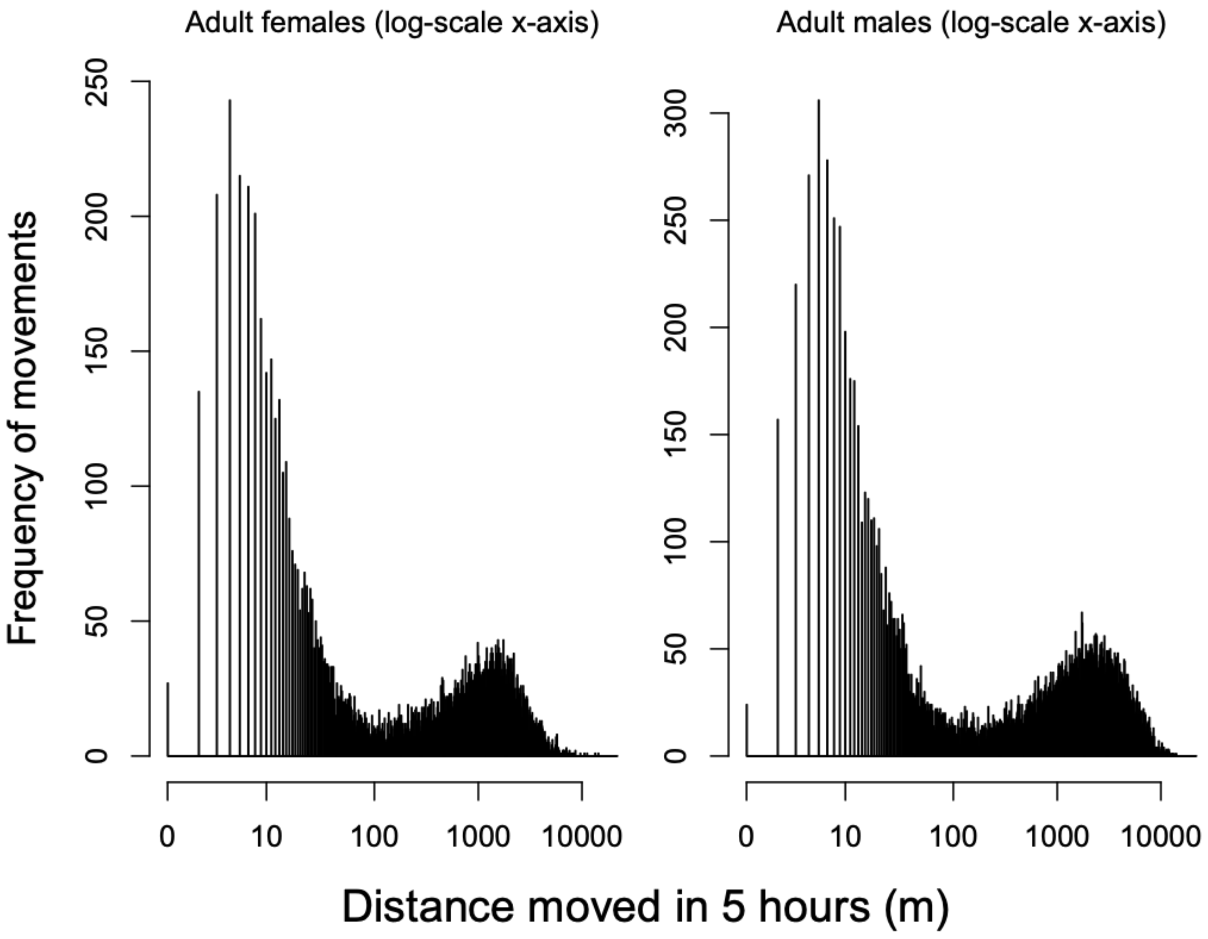

Fig. S2. Histogram of the log-transformed raw snow leopard GPS-movement data showing the frequency of observed snow leopard movements related to the straight-line distance (displacement) they moved from one GPS position to the next (5 hours later). The data have been separated into females (left panel) and males (right panel).

Supplementary Figure S3. Histogram of the log transformed ibex data

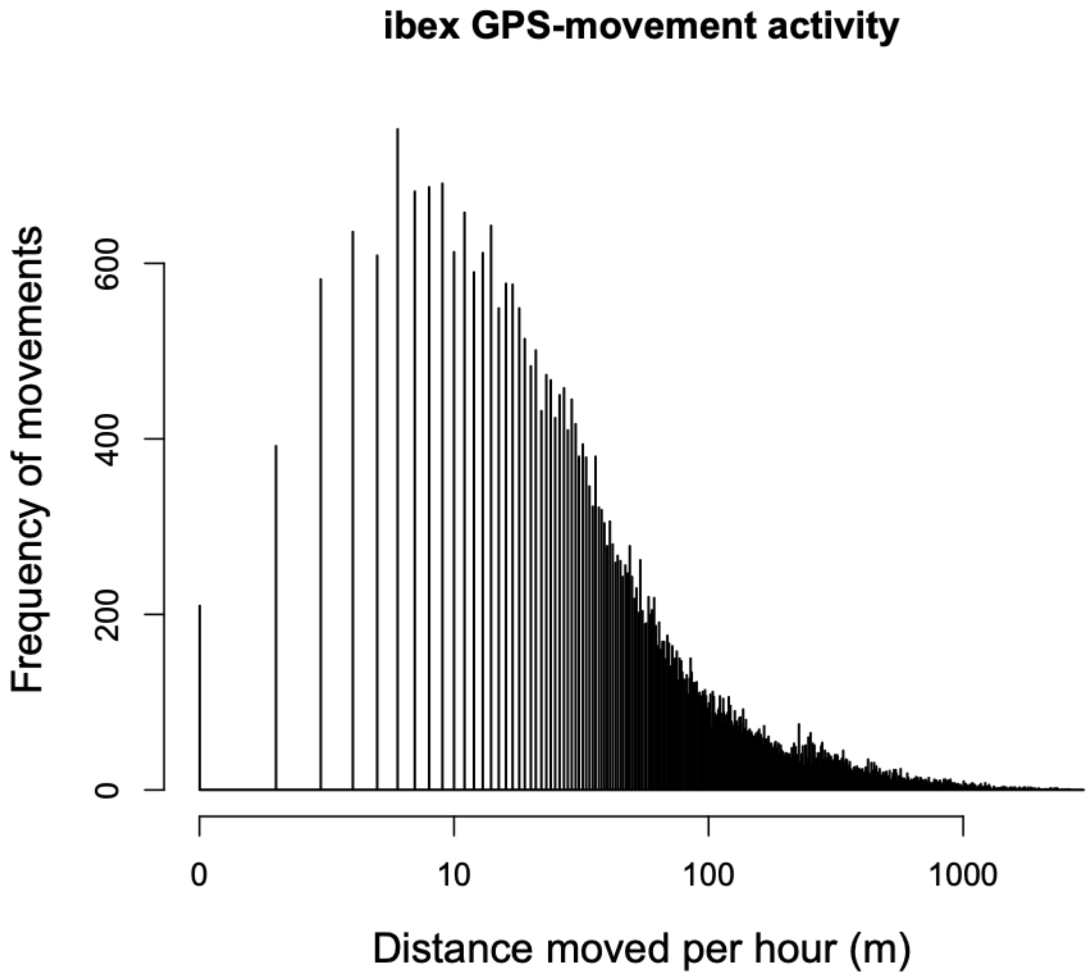

Fig. S3. Histogram of the log-transformed raw ibex GPS-movement data showing the frequency of observed ibex movements related to the straight-line distance (displacement) they moved from one GPS position to the next (1 hour later).

Supplementary Figure S4. Histogram of the log transformed domestic goat data

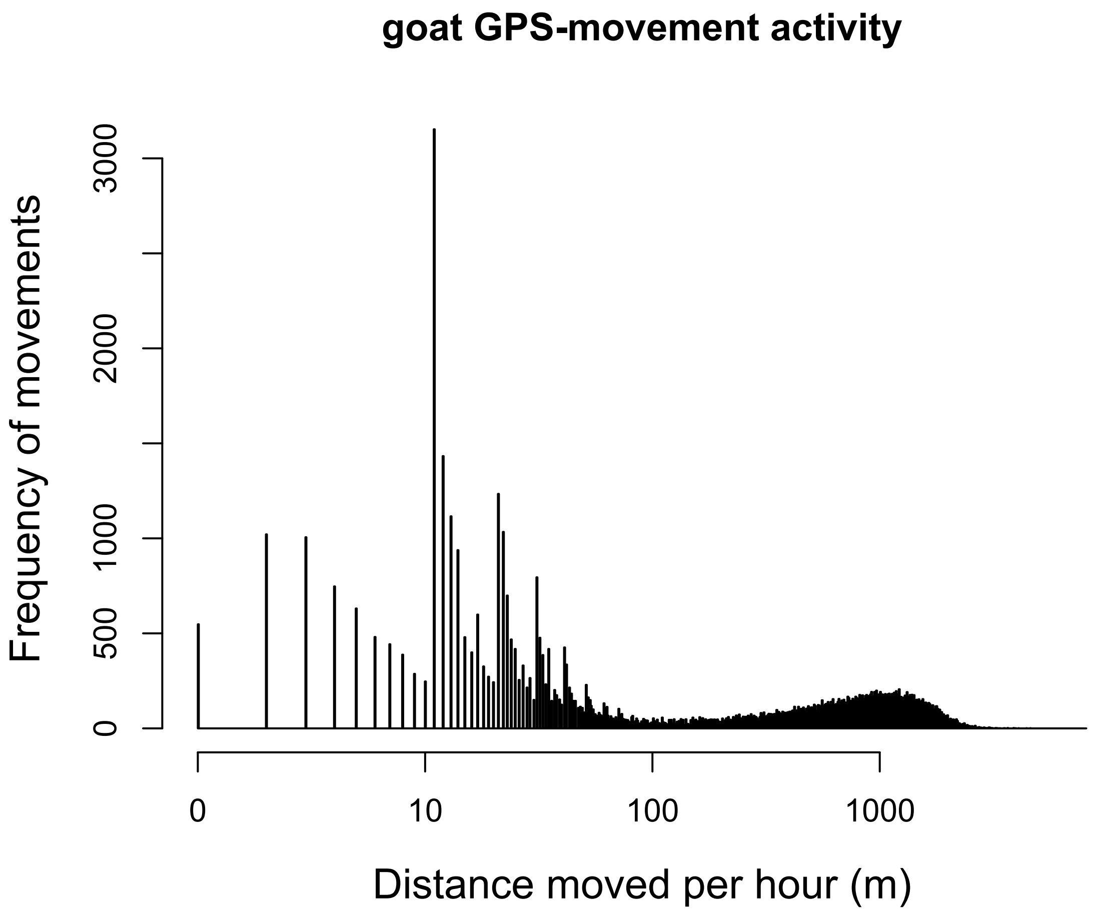

Fig. S4. Histogram of the log-transformed raw goat GPS-movement data showing the frequency of observed goat movements related to the straight-line distance (displacement) they moved from one GPS position to the next (1 hour later).

Supplementary Figure S5. Sex disaggregated snow leopard activity data

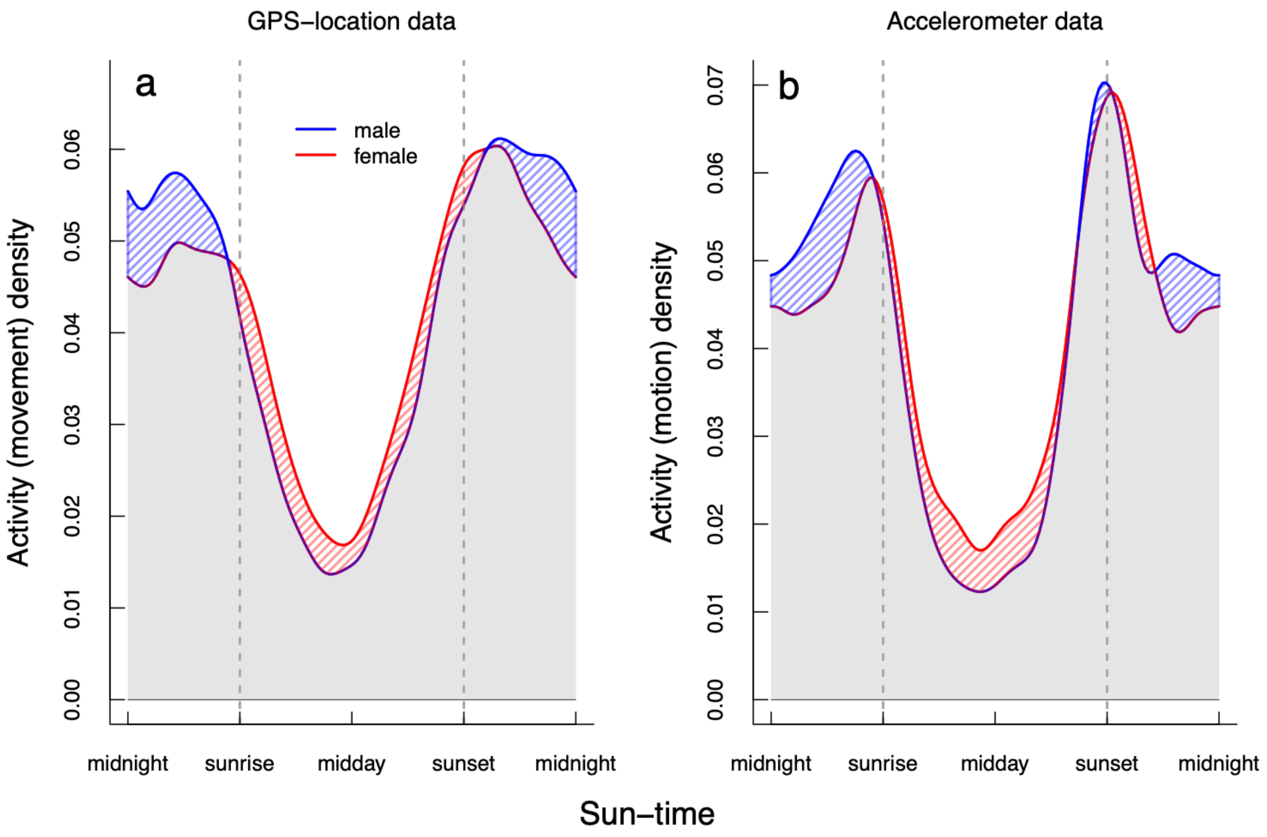

Fig. S5. The relative proportion of snow leopard activity across the 24-hour cycle for males (blue) and females (red) for the two types of activity data collected from GPS-collars (a = GPS displacement movement data; b = accelerometer motion activity data). The activity densities on the y-axis have no absolute meaning, but rather are relative measures of probability density calculated from the raw movement data. The time of all observations has been standardized along the x-axis using 'sun-times'; where the time of observation on each day is calibrated to sunrise, solar-noon (midday) and sunset. These overlap plots show the activity overlap between males and female (grey) and highlight periods when males or females have greater activity (blue or red shaded, respectively).

Supplementary Figure S6. Nocturnal SL motion activity relative to moon illumination

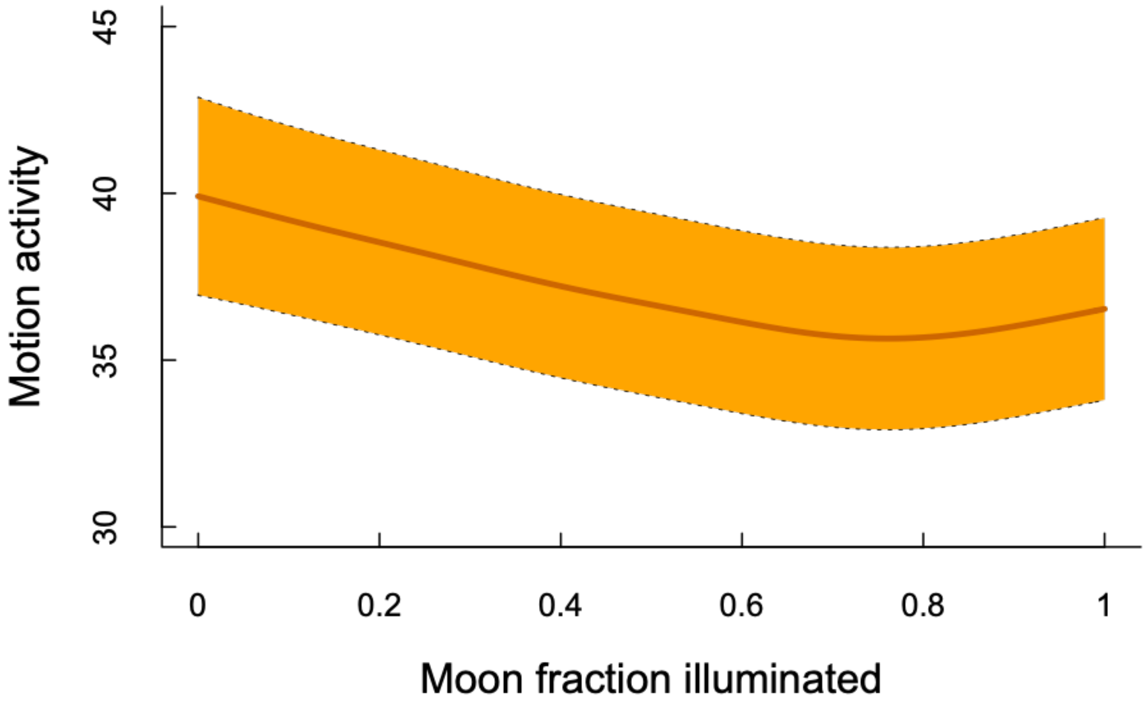

Fig. S6. Prediction of snow leopard accelerometer activity level based on the fraction of the moon being illuminated (where 0 = no moon, 1 = full moon) from a GAMM using moon fraction as a fixed effect smoother and individual ID as a random effect. The line is the predicted mean and the shaded area is the standard error of the prediction.
